# Evaluation of Neonatal Services Provided in a Level II NICU Utilizing Hybrid Telemedicine: A Prospective Study

**DOI:** 10.1089/tmj.2018.0262

**Published:** 2020-02-05

**Authors:** Abhishek Makkar, Mike McCoy, Gene Hallford, Arlen Foulks, Michael Anderson, Jennifer Milam, Marla Wehrer, Erica Doerfler, Edgardo Szyld

**Affiliations:** Division of Newborn Medicine, Department of Pediatrics, University of Oklahoma Health Sciences Center, Oklahoma City, Oklahoma.

**Keywords:** telemedicine, pediatrics, e-health, telehealth, telecommunications, NICU

## Abstract

***Objective:*** To evaluate the safety and efficacy of premature infant treatment managed by hybrid telemedicine versus conventional care.

***Methods:*** Prospective, noninferiority study comparing outcomes of premature infants at Comanche County Memorial Hospital's (CCMH) Level II neonatal intensive care unit (NICU) with outcomes at OU Medical Center's (OUMC) Level IV NICU. All 32–35 weeks gestational age (GA) infants admitted between May 2015 and October 2017 were included. Infants requiring mechanical ventilation >24 h or advanced subspecialty care were excluded. Outcome variables were: length of stay (LOS), respiratory support, and time to full per oral (PO) feeds. Parents at both centers were surveyed about their satisfaction with the care provided. Between-group comparisons were performed by using Chi-square or Fisher's exact test. LOS was assessed for normality by using the Shapiro–Wilk test, and robust regression was used to construct a multivariable regression model to test the independent effect of location on LOS. All analyses were performed by using SAS v. 9.3 (SAS Institute, Cary, NC).

***Results:*** Data from 85 CCMH and 70 OUMC neonates were analyzed. CCMH neonates had significantly shorter LOS, reached full PO feeds sooner, and had fewer noninvasive ventilation support days. Location had a significant independent effect (p = 0.001) on LOS while controlling for GA, gender, race, surfactant use, inborn/outborn status, and 5-min APGAR scores. CCMH patients had reduced LOS of 3.01 days (95% confidence interval 1.1–4.8) than OUMC patients. Eighty-five surveys at CCMH and 66 at OUMC were analyzed. Compared with CCMH, OUMC parents reported more travel distance difficulties. 92.5% reported telemedicine experience as good or excellent, whereas 1.5% reported it as poor.

***Conclusion(s):*** Hybrid telemedicine is a safe and effective way to extend intensive neonatal care to medically underserved areas. Parental satisfaction with use of hybrid telemedicine is high and comparable to conventional care.

## Introduction

The preterm birth rate in the United States increased to 9.85% in 2016, a 3% rise from 2014 (9.57%). Most of this increase was among infants born late preterm, up from 6.87% to 7.09%.^1^ Although most hospitals have facilities to deliver babies, only a few have the capability to provide specialized care to neonates.^2^

The concept of perinatal regionalization was introduced a few decades ago in an effort to provide high-quality, risk-appropriate care to the mother–infant dyad. It is unfortunate that the evidence basis for implementing the perinatal regionalized system has lagged behind the development of such systems.^3^ Since then, several studies have demonstrated decreased mortality rates among very low-birth-weight infants who are delivered and treated at higher levels of neonatal care (Level III or higher).^4–8^ The effect of perinatal regionalization on late preterm infants is not known. It is unclear where moderately and late preterm infants should receive their neonatal care.^9^

The American Academy of Pediatrics has published guidelines defining the levels of neonatal care and required personnel. These guidelines added in-house neonatal services to the scope of Level II neonatal intensive care units (NICUs).^10^ Unavailability of neonatologists poses a challenge to the effective implementation of these guidelines. A recently published descriptive analysis of current perinatal resources showed that there are 12.0 neonatologists per 10,000 live births in the United States. Oklahoma is 1 of the 13 states in which less than 80% of women of reproductive age have access (defined as living within 50 miles) to an NICU.^11^ Consequently, many neonates, including moderately ill late preterm infants, are often transferred to regional Level IV NICUs. This results in mother–infant separation and potential breastfeeding disruption, high transportation costs, the emotional costs of limited visitation, and family stress. Therefore, there has been a recent interest in utilizing alternatives to patient transport for coordination of patient care. One promising alternative is the use of technology, such as telemedicine, which could allow patients to remain at a local, less specialized center and still have access to a physician with subspecialty expertise.

Telemedicine use in critical care settings is mostly limited to pediatric and adult populations.^12–15^ The use of telemedicine in the neonatal population is limited to consultation, teleechocardiography, retinopathy of prematurity screening, neonatal resuscitation program education, postdischarge follow-up, and family involvement.^16–22^ To date, few studies report telemedicine use as a primary means of providing care to premature infants in the NICU.^23,24^ Our current prospective study was designed to evaluate the safety and efficacy of the treatment of premature infants managed by a hybrid telemedicine system in a satellite Level II NICU at Comanche County Memorial Hospital (CCMH) in Lawton, Oklahoma, compared with conventional management provided to a similar population in a regional Level IV NICU at OU Medical Center (OUMC) in Oklahoma City.

## Methods

This study is a prospective noninferiority design that was conducted in NICUs at OUMC and CCMH. OUMC is a tertiary care facility with a Level IV NICU (92 beds) that provides continuous intensivist coverage, including daily bedside rounds. CCMH is a community-level medical facility, located 90 miles southwest to OUMC that offers Level II NICU care (8 beds). Infants in the CCMH NICU are managed by using a hybrid telemedicine system. Regulatory approval was separately obtained through the Institutional Review Board at the University of Oklahoma Health Sciences Center (OUHSC) for OUMC and the internal research committee for CCMH, Lawton.

Neonates between 32 and 35 weeks gestational age (GA), admitted to either CCMH or OUMC between May 2015 and October 2017, were included. To ensure that patients at these units were comparable in all respects other than travel distance, only families living >1 h away were included at OUMC and only families living <1 h away were included at CCMH. This was done to have an ideal control population at OUMC that was geographically similar to the population at satellite NICU and to get the true effect of telemedicine use on parental satisfaction.

Those neonates needing more than 24 h of mechanical ventilation or requiring advanced subspecialty care were excluded. Also, patients who were transferred between CCMH and OUMC were excluded due to logistical issues as the study was looking at a comparison of care between two centers.

Distance health care services were made available to CCMH patients by using a telemedicine communication system meeting the stringent clinical practice guidelines of the American Telemedicine Association.^25^ This complex communication system included a Polycom^®^ (Pleasanton, CA) videoconferencing base (HDX 7000) directly connected to the CCMH hospital server. This server was configured to meet federally mandated Health Insurance Portability and Accountability Act (HIPAA) requirements. Between-site communication was accomplished by using secure fiber optic lines and routed through a secure, HIPAA-compliant network located at OUHSC. These fiber optic lines were direct dedicated lines utilized as a dedicated link to the hospital server at CCMH, from where the signal was transmitted through a secure fiber optic connection to the campus at OUMC and routed through the OUMC secure network to the individual neonatologist's secure laptop or desktop. The Polycom unit was configured to support high-definition, two-way audiovisual signals by using a high-speed Ethernet connection.

Before each telemedicine session, neonatologists were contacted by a neonatal nurse practitioner (NNP) physically located at CCMH. Once contacted, the remote intensivist at OUMC initiated a videoconference call by using the Polycom interface. The NNP then accepted input from the system, which initiates full operation mode. The mobile telemedicine cart was then moved from bed-to-bed as the off-site provider and local NNP conducted bedside rounds. The NNP physically present at the bedside then presented relevant patient information to the remote intensivist at OUMC, including information on all impacted organ systems, as part of the standard rounding practice. At this time, the off-site physician, assisted by an NNP, examined the infant via the telemedicine cart and participated by asking questions and making clinical recommendations as needed. The team then devised a plan for the day for each patient. As is standard of care, either during typical in-person rounds or during rounds conducted via telemedicine, parents are encouraged to attend and actively participate in these daily patient visits. In addition to using the telemedicine system for daily rounding, the NNP may use alternate means of contacting the intensivist as questions or issues arise. These alternative communication avenues include telephone, text, or e-mail message. Daily plan for the patients is documented by the NNP in the Electronic Medical Record system on a daily basis and is signed out to the incoming NNP during handover report. Physicians have remote access to NNP documentation.

Primary outcome measures included patients' length of stay (LOS), type and duration of respiratory support, and time to full per oral (PO) feedings measured in days. Data were collected by chart review. The LOS was reflective of total hospital stay, as both centers have infants rooming in the day before discharge as part of their standard practice. Comparisons were done by stratifying GA in three groups: 32 weeks (32–32 6/7), 33 weeks (33–33 6/7), and 34 weeks (34–35 0/7). Between-group comparisons were performed by using Chi-square or Fisher's exact test, as appropriate for the type of data analyzed. The LOS was assessed for normality by using the Shapiro-Wilk test. Robust regression was used to construct a multivariable regression model to test the independent effect of location on LOS while controlling for GA, gender, race, surfactant use, inborn/outborn status, and 5-min Apgar scores. Apgar scores at 1 min were excluded due to the high correlation with Apgar scores at 5 min (0.75, *p* ≤ 0.0001). Surfactant use was used as a surrogate to match for severe respiratory distress. Chi-square or Fisher's exact test was used with a significance level of 0.05. All analyses were carried out by using SAS v. 9.3 (SAS Institute, Cary, NC).

Previous experience with this population indicated and expected mean LOS of 15 days with a standard deviation of 4 days.^24^ A sample of 230 (115 telemedicine, 115 reference) will have 80% power to establish noninferiority in LOS for the telemedicine group by using a noninferiority margin of 0.1, which indicated a 10% difference in LOS.

**Fig. 1.**
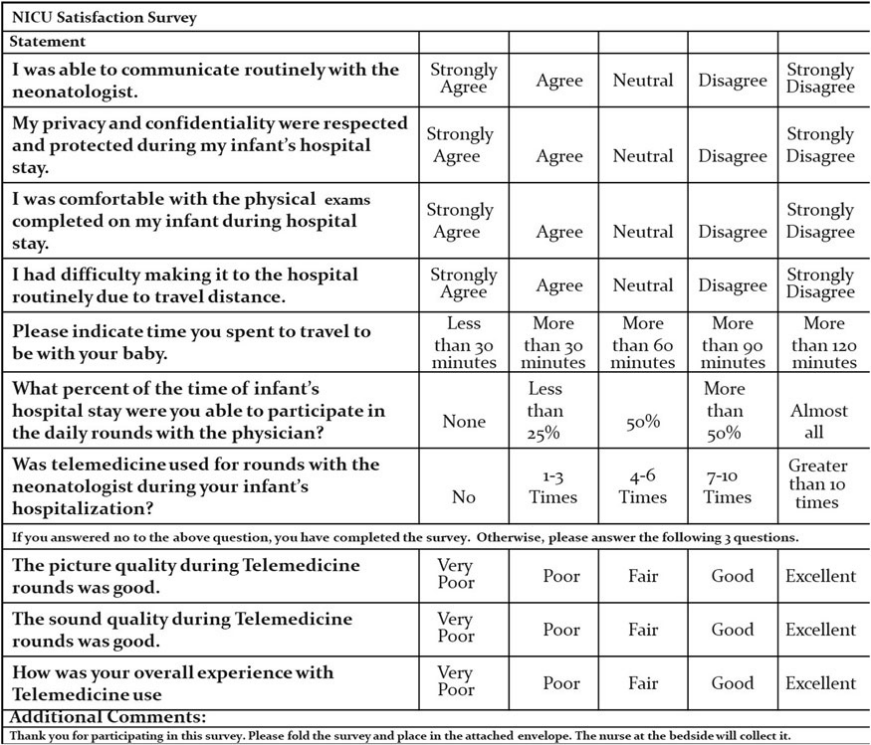
NICU satisfaction survey. NICU, neonatal intensive care unit.

Parents at both centers were surveyed about their satisfaction with the care provided by utilizing a 10-question survey (*Fig. 1*). The final three survey questions addressed satisfaction with the technical aspects of telemedicine and were answered only by parents who were exposed to telemedicine during their stay. The survey consisted of five-point Likert scale questions with response options of strongly agree, agree, neutral, disagree, and strongly disagree. The survey was given to parents before discharge.

In addition, transport cost savings were calculated by multiplying the total number of study patients at the satellite Level II NICU by standard transport cost per patient. The transport cost per patient was calculated as Base Cost ($3,352) + Mileage Cost ($23.91 per nautical mile × distance between the two hospitals). Base and mileage costs were for Rotor transport by company Air Methods^®^, Colorado.

## Results

**Fig. 2.**
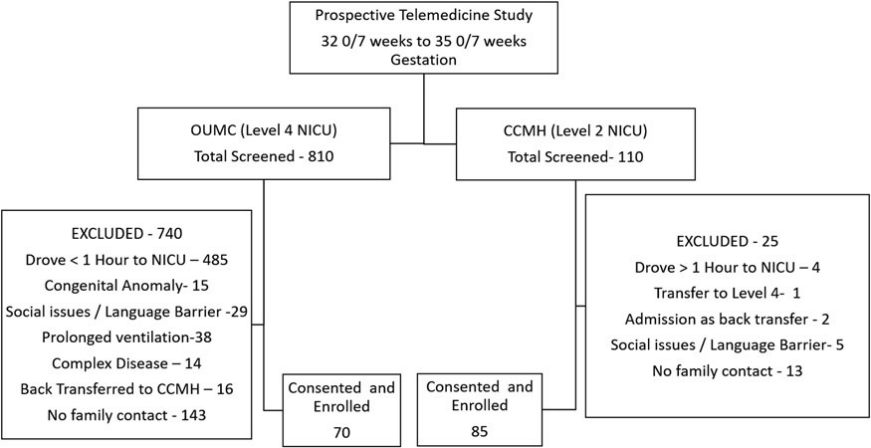
Study population flowchart diagram. CCMH, Comanche County Memorial Hospital; NICU, neonatal intensive care unit; OUMC, Oklahoma University Medical Center.

**Fig. 3.**
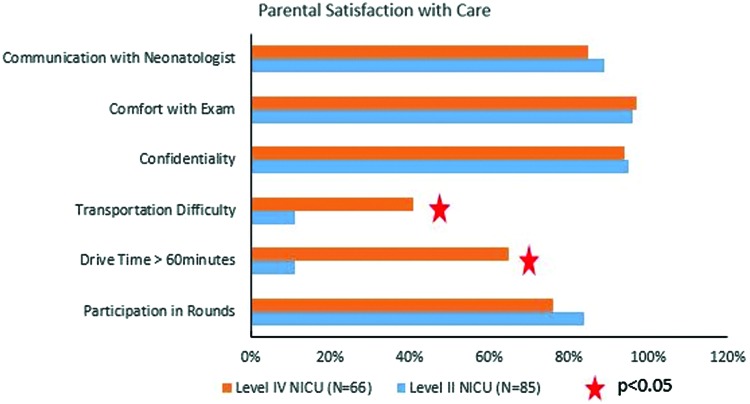
Parental satisfaction with care at both centers. NICU, neonatal intensive care unit. Color images are available online.

**Fig. 4.**
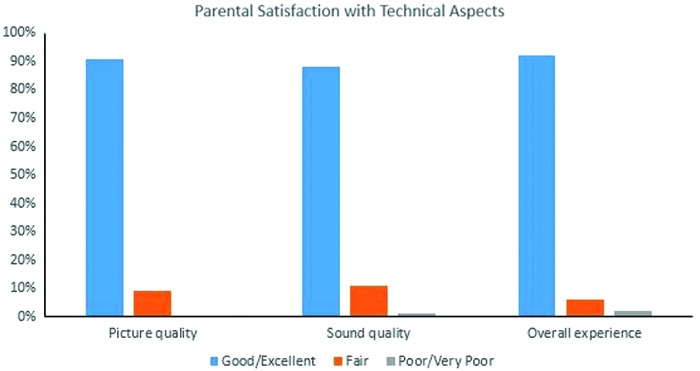
Parental satisfaction with technical aspects of telemedicine. Color images are available online.

Study population flowchart diagram is demonstrated in *Figure 2*. At the interim analysis, primary outcome measure was statistically significantly shorter at CCMH compared with OUMC ensuring noninferiority conclusion; therefore, the study was stopped at that point. One hundred fifty-five neonates, 85 at CCMH and 70 at OUMC, met inclusion/exclusion criteria and were included in the analysis. Approximately 28% of CCMH neonates were less than 33 weeks GA, compared with 34% of neonates at OUMC. *Table 1* The demographic characteristics of these groups are provided in *Table 2*. About 92% of enrolled infants at CCMH were inborn, compared with 80% at OUMC. Prematurity, hyperbilirubinemia, respiratory distress syndrome, and hypoglycemia were the four most common diagnoses at both centers. CCMH neonates had significantly shorter hospital stays, reached full PO feeds sooner, and required fewer days on noninvasive ventilation support (*Table 3*). LOS was not normally distributed, so the multivariable regression model was created by using robust regression methods. Location had a significant independent effect (*p* = 0.001) on the LOS while controlling for GA, race, gender, severe respiratory distress syndrome or surfactant use, 5-min Apgar scores, and inborn/outborn status. Specifically, participants at CCMH had a reduced LOS of 3.01 days (95% confidence interval 1.1–4.8) compared with those at OUMC. *Table 4* Data from 85 surveys at CCMH and 66 at OUMC were analyzed. Compared with CCMH patients, OUMC parents reported more travel difficulties, and they had lower participation in rounds (*Fig. 3*). Participation in rounds was also used as a surrogate measure for parental visitation. Overall, 92.5% of the CCMH parents reported the telemedicine experience as good or excellent, whereas 1.5% reported it as poor (*Fig. 4*). Lastly, we estimated that the transport cost savings were $475,000 [no. of patients enrolled in the study at satellite Level II NICU (85) × The transport cost per patient: Base Cost ($3,352) + Mileage Cost ($23.91 per nautical mile × 91 miles)].

**Table 1.** Number of Neonates Stratified by Gestational Age for Each Center

GA (WEEKS)	CCMH, N (%)	OUMC, N (%)	p
32	11 (12.94)	12 (17.14)	0.1345
33	17 (20.00)	22 (31.43)	
34	57 (67.06)	36 (51.43)	
Total	85	70	

CCMH, Comanche County Memorial Hospital; GA, gestational age; OUMC, Oklahoma University Medical Center.

**Table 2.** Demographic Characteristics of Patients at Each Center

PARAMETER	CCMH, N (%)	OUMC, N (%)	p
Race			
African American	21 (24.71)	5 (5.71)	0.0015^*^
American Indian	6 (7.06)	5 (7.14)	
Hispanic	6 (7.06)	3 (4.29)	
Caucasian	36 (42.35)	50 (71.43)	
Others	16 (18.83)	8 (11.43)	
Gender			
Male	55 (65)	39 (56)	0.2541
Female	30 (35)	31 (44)	
Birth location			
Inborn	78 (92)	56 (80)	0.0332^*^
Outborn	7 (8)	14 (20)	

^*^ *p* < 0.05.

CCMH, Comanche County Memorial Hospital; OUMC, Oklahoma University Medical Center.

**Table 3.** Outcome Measures

GA (WEEKS) (CCMH/OUMC) (N)	CCMH, MEAN (SD)	OUMC, MEAN (SD)	p
LOS (days)			
32 Weeks (11/12)	17.55 (4.82)	19.75 (4.11)	0.2503
33 Weeks (17/22)	12.71 (4.37)	16.05 (4.40)	0.0182^*^
34 Weeks (57/36)	9.63 (4.36)	13.39 (6.56)	0.0053^*^
Overall average (85/70)	11.27 (5.15)	15.31 (6.00)	<0.0001^*^
Days on noninvasive ventilation			
32 Weeks (9/6)	1.81 (1.13)	4.8 (4.95)	1.0000
33 Weeks (11/13)	2.54 (2.94)	3.41 (3.26)	0.4869
34 Weeks (32/13)	2.18 (1.93)	4.61 (8.16)	0.2442
Overall average (52/32)	2.20 (2.05)	4.16 (5.85)	0.1685
Days on oxygen			
32 Weeks (6/5)	6.76 (11.27)	4.49 (3.93)	1.0000
33 Weeks (7/11)	2.67 (3.32)	2.04 (3.93)	0.0853
34 Weeks (23/10)	2.59 (1.99)	4.99 (7.12)	0.9844
Overall average (36/26)	3.3 (5)	3.65 (5.37)	0.1967
Time to reach full feeds (days)			
32 Weeks (7/11)	4.57 (2.23)	5.50 (1.75)	0.3613
33 Weeks (11/22)	8.82 (18.36)	5.00 (3.12)	0.2819
34 Weeks (36/27)	3.72 (1.92)	4.00 (2.39)	0.6364
Overall average (54/60)	4.87 (8.41)	4.63 (2.62)	0.0853
Time to reach full PO feeds (days)			
32 Weeks (11/12)	9.73 (7.54)	17.5 (4.66)	0.0068^*^
33 Weeks (17/22)	9.94 (9.67)	12.86 (5.37)	0.0083^*^
34 Weeks (57/36)	6.42 (4.56)	10.03 (8.95)	0.0624
Overall average (83/70)	7.43 (6.30)	12.20 (7.77)	<0.0001^*^

^*^ *p* < 0.05.

CCMH, Comanche County Memorial Hospital; GA, gestational age; LOS, length of stay; OUMC, Oklahoma University Medical Center; PO, per oral; SD, standard deviation.

**Table 4.** Multiple Linear Regression for Length of Stay

EXPLANATORY VARIABLES	DF	ESTIMATE	SE	95% CI	CHI-SQUARE		Pr > ChiSq
Intercept	1	122.1797	19.0883	84.7673	159.5920	40.97	<0.0001
Gestational weeks	1	−3.2315	0.5716	−4.3518	−2.1112	31.96	<0.0001
Race	6						NS
Sex							
Female	1	0.0933	0.9037	−1.6780	1.8646	0.01	0.9178
Male	0	Reference					
Inborn or outborn							
Inborn		0.8774	1.3149	−1.6998	3.4546	0.45	0.5046
Outborn	0	Reference					
APGAR score at 5 min	1	0.2829	0.3613	−0.4253	0.9911	0.61	0.4337
Location							
CCMH	1	−3.0156	0.9491	−4.8758	−1.1555	10.10	0.0015
OUMC	0	Reference					
Surfactant dose							
No	1	−2.0807	1.2058	−4.4439	0.2826	2.98	0.0844
Yes	0	Reference					
Scale	1	4.2379					

CCMH, Comanche County Memorial Hospital; CI, confidence interval; OUMC, Oklahoma University Medical Center.

## Discussion

This prospective study results showed that patient outcome measures of infants managed by the hybrid telemedicine system at a satellite Level II NICU were not inferior to those of conventional management provided to similar infants at a regional Level IV NICU. Parents also reported a high level of satisfaction with technical aspects of telemedicine. There were no differences noted in the degree of parental satisfaction with the other aspects of care when the care was provided by using telemedicine versus conventional care. LOS was the main primary outcome measure and was noted to be shorter for infants who received care at the satellite Level II NICU via telemedicine than for similar infants who received care at the regional Level IV NICU.

A few factors may result in the shorter LOS at this satellite Level II NICU. First, this hybrid telemedicine setup allowed families to spend more time with their babies due to fewer travel difficulties, as infants were managed at a local hospital closer to home. Higher family participation permitted longer mother–infant bonding, which likely was an influencing factor for the infant's ability to reach full PO feedings sooner, therefore necessitating a shorter LOS. Second, we speculate that nursing assignments at the Level IV NICU could be another reason. There are times when the nursing assignment at the Level IV NICU could include taking care of a high acuity patient at the same time as another patient who is a “feeder and grower.” This approach could result in less attention, from a nursing standpoint, to work on the growth and discharge planning needs of the late premature infant. The lower acuity assignments at Level II permit nursing to work on the growth and discharge planning needs of late premature infants.

In a recently published study, our group reported similar favorable outcome measures with telemedicine use.^24^ Our previous study was retrospective and did not assess parental satisfaction or transport cost savings. To our knowledge, there is only one reported prospective trial on the use of telemedicine for rounding in NICU.^23^ This study demonstrated that telemedicine can be utilized by neonatologists to perform daily patient rounds in the neonatal intensive care unit as long as direct bedside care providers are available. It was conducted at an academic institution where there was 24/7 access to a resident and neonatologist, if needed. The neonatologist was also available in-house at night. This feasibility study tested whether telemedicine rounds are effective and showed that there were no significant differences in outcome measures when telemedicine care was utilized. The authors concluded that although they do not envision the use of this system in academic settings where neonatologists are present 24/7, the technology has clear potential in settings where there is a lack of access to neonatologists in person.

Bourque and Hwang recently wrote an interesting summary of the utilization of NICU care among infants of varying birth weights and GAs.^9^ The author stated that there is sufficient evidence that very low-birth-weight infants have better outcomes when they are delivered and treated at higher levels of neonatal care (Level III/IV), but less is known about where moderately and late preterm infants should receive their neonatal care. Harrison et al. also reported that there the NICU is underused for very low-birth-weight infants and overused for infants >1,500 g, based on NICU bed supply.^26^ To date, there are no studies comparing the outcome measures of care provided to late premature infants at Level II versus Level IV NICUs.

Although our results were reassuring, the study has some limitations. One such limitation was the small sample size. Although the power analysis called for the enrollment of 230 study participants, the inclusion/exclusion criteria resulted in only 155 neonates being eligible for inclusion in the data analysis. Given that at the interim analysis, primary outcome measure was statistically significantly shorter at CCMH compared with OUMC ensuring noninferiority conclusion, we did not feel that we should further extend the study in the hopes of meeting this number. Future studies will need to be done, with a larger subject sample, to fully address this issue. Due to the nature of the study, we were not able to randomize patients. However, we made attempts to match for acuity to compare medically similar patients at both centers, utilizing stringent inclusion/exclusion criteria. Based on transport cost savings, we concluded that the system is cost effective. However, we did not evaluate detailed hospital costs per site.

Our next objective is to do a detailed cost analysis to report the financial feasibility of the implementation of this system. We speculate that the reduced LOS would result in significant hospital cost savings.

## Conclusions

We conclude that this hybrid telemedicine system is a safe and cost-effective way to extend intensive care to late premature neonates in medically underserved areas. Parental satisfaction with the use of hybrid telemedicine was particularly high, due to reductions in transportation difficulties, as the infants were able to stay at a local hospital without compromising the quality of care.

## References

[1] Martin JA, Hamilton BE, Osterman MJK, Driscoll AK, Drake P Births: Final Data for 2016. Natl Vital Stat Rep 2018**;** 67:1–55 29775434

[2] Wang HE, Yealy DM Distribution of specialized care centers in the United States. Ann Emerg Med 2012**;** 60:632–637 e7. 22633341 10.1016/j.annemergmed.2012.02.020

[3] Lorch SA, Myers S, Carr B The regionalization of pediatric health care. Pediatrics 2010**;** 126:1182–1190 21041285 10.1542/peds.2010-1119 PMC3915403

[4] Paneth N, Kiely JL, Wallenstein S, Susser M The choice of place of delivery. Effect of hospital level on mortality in all singleton births in New York City. Am J Dis Child 1987**;** 141:60–64 3788883 10.1001/archpedi.1987.04460010060024

[5] Gortmaker S, Sobol A, Clark C, Walker DK, Geronimus A The survival of very low-birth weight infants by level of hospital of birth: A population study of perinatal systems in four states. Am J Obstet Gynecol 1985**;** 152:517–524 4014345 10.1016/0002-9378(85)90618-0

[6] Kamath BD, Box TL, Simpson M, Hernandez JA Infants born at the threshold of viability in relation to neonatal mortality: Colorado, 1991 to 2003. J Perinatol 2008**;** 28:354–360 18273030 10.1038/sj.jp.7211918 PMC3612026

[7] Menard MK, Liu Q, Holgren EA, Sappenfield WM Neonatal mortality for very low birth weight deliveries in South Carolina by level of hospital perinatal service. Am J Obstet Gynecol 1998**;** 179:374–381 9731841 10.1016/s0002-9378(98)70367-9

[8] Dooley SL, Freels SA, Turnock BJ Quality assessment of perinatal regionalization by multivariate analysis: Illinois, 1991–1993. Obstet Gynecol 1997**;** 89:193–198 9015019 10.1016/S0029-7844(96)00450-4

[9] Bourque SL, Hwang SS Underuse versus overuse of neonatal intensive care: What is the right amount? J Pediatr 2018**;** 192:5–6 29126596 10.1016/j.jpeds.2017.09.048

[10] American Academy of Pediatrics Committee on Fetus and Newborn. Levels of neonatal care. Pediatrics 2012**;** 130:587–597 22926177 10.1542/peds.2012-1999

[11] Brantley MD, Davis NL, Goodman DA, Callaghan WM, Barfield WD Perinatal regionalization: A geospatial view of perinatal critical care, United States, 2010–2013. Am J Obstet Gynecol 2017**;** 216:185 e1–e10. 27773712 10.1016/j.ajog.2016.10.011 PMC11289569

[12] Munoz RA, Burbano NH, Motoa MV, Santiago G, Klevemann M, Casilli J Telemedicine in pediatric cardiac critical care. Telemed J E Health 2012**;** 18:132–136 22283363 10.1089/tmj.2011.0090

[13] Rosenfeld BA, Dorman T, Breslow MJ, Pronovost P, Jenckes M, Zhang N, et al. Intensive care unit telemedicine: Alternate paradigm for providing continuous intensivist care. Crit Care Med 2000**;** 28:3925–3931 11153637 10.1097/00003246-200012000-00034

[14] Willmitch B, Golembeski S, Kim SS, Nelson LD, Gidel L Clinical outcomes after telemedicine intensive care unit implementation. Crit Care Med 2012**;** 40:450–454 22020235 10.1097/CCM.0b013e318232d694

[15] Binder WJ, Cook JL, Gramze N, Airhart S Telemedicine in the intensive care unit: Improved access to care at what cost? Crit Care Nurs Clin North Am 2018**;** 30:289–296 29724446 10.1016/j.cnc.2018.02.010

[16] Wenger TL, Gerdes J, Taub K, Swarr DT, Deardorff MA, Abend NS Telemedicine for genetic and neurologic evaluation in the neonatal intensive care unit. J Perinatol 2014**;** 34:234–240 24406740 10.1038/jp.2013.159 PMC3943754

[17] Fang JL, Collura CA, Johnson RV, Asay GF, Carey WA, Derleth DP, et al. Emergency video telemedicine consultation for newborn resuscitations: The Mayo Clinic Experience. Mayo Clin Proc 2016**;** 91:1735–1743 27887680 10.1016/j.mayocp.2016.08.006

[18] Moser L, Diogenes T, Mourato FA, Mattos S Learning echocardiography and changing realities through telemedicine. Med Educ 2014**;** 48:1125–1126 25307664 10.1111/medu.12561

[19] Wang SK, Callaway NF, Wallenstein MB, Henderson MT, Leng T, Moshfeghi DM SUNDROP: Six years of screening for retinopathy of prematurity with telemedicine. Can J Ophthalmol 2015**;** 50:101–106 25863848 10.1016/j.jcjo.2014.11.005

[20] Jain A, Agarwal R, Chawla D, Paul V, Deorari A. Tele-education vs classroom training of neonatal resuscitation: A randomized trial. J Perinatol 2010**;** 30:773–779 20357810 10.1038/jp.2010.42

[21] Willard A, Brown E, Masten M, Brant M, Pouppirt N, Moran K, et al. Complex surgical infants benefit from postdischarge telemedicine visits. Adv Neonatal Care 2018**;** 18:22–30 29373346 10.1097/ANC.0000000000000460

[22] Gray J, Jones PC, Phillips M, Gertman P, Veroff D, Safran C. Telematics in the neonatal ICU and beyond: Improving care for high-risk newborns and their families. Proc AMIA Annu Fall Symp 1997**:** 413–417 9357659 PMC2233440

[23] Garingo A, Friedlich P, Chavez T, Tesoriero L, Patil S, Jackson P, et al. “Tele-rounding” with a remotely controlled mobile robot in the neonatal intensive care unit. J Telemed Telecare 2016**;** 22:132–138 26116855 10.1177/1357633X15589478

[24] Makkar A, McCoy M, Hallford G, Escobedo M, Szyld E A hybrid form of telemedicine: A unique way to extend intensive care service to neonates in medically underserved areas. Telemed J E Health 2018**;** 24:717–721 29298407 10.1089/tmj.2017.0155

[25] Krupinski EA. Core operational guidelines for telehealth services involving provider-patient interactions: American Telemedicine Association; 2014. Available at http://etelmed.com/wp-content/uploads/2014/03/Core-Operational-Guidelines-for-Telehealth-Services.pdf (last accessed 111, 2019).

[26] Harrison WN, Wasserman JR, Goodman DC Regional variation in neonatal intensive care admissions and the relationship to bed supply. J Pediatr 2018**;** 192:73–79 e4. 28969888 10.1016/j.jpeds.2017.08.028

